# Supplementary material for: Structural insights into the π-π-π stacking mechanism and DNA-binding activity of the YEATS domain
Source: Nat Commun. 2018 Nov 1;9:4574. doi: 10.1038/s41467-018-07072-6 (PMC6212594; doi:10.1038/s41467-018-07072-6)
Supplement: Supplementary file 1 — Supplementary Information [file 41467_2018_7072_MOESM1_ESM.pdf]

Supplementary Information

Structural insights into the π-π-π stacking mechanism and DNA-binding activity of the YEATS domain

Brianna J. Klein, Kendra R. Vann, Forest H. Andrews, et al.,

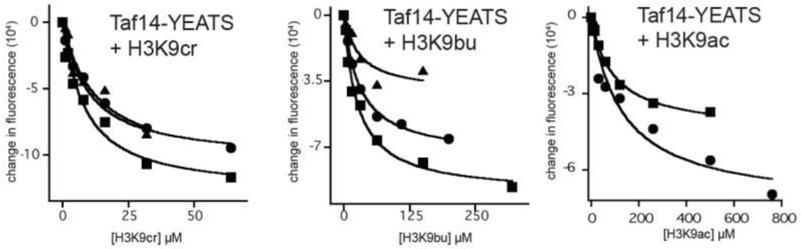

Supplementary Figure 1. Binding curves used to determine K_d values of Taf14-YEATS by tryptophan fluorescence. Related to Figure 1.

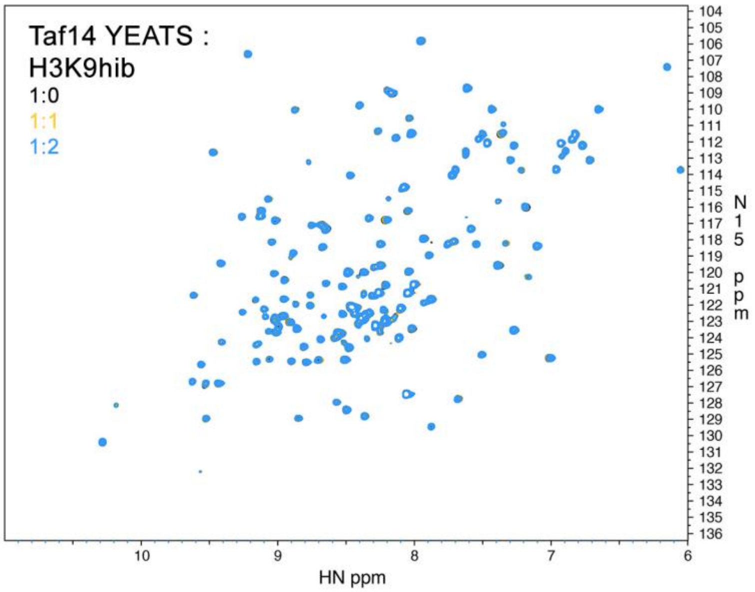

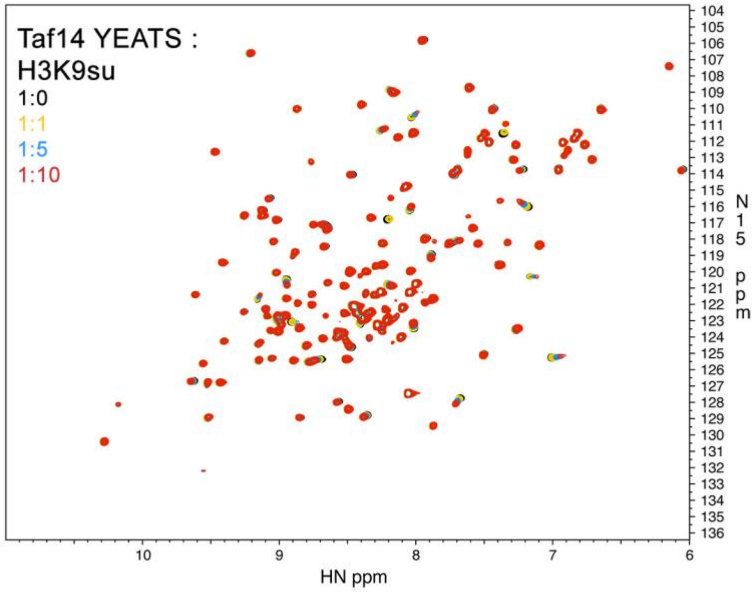

Supplementary Figure 2. Superimposed $^{1}H,^{15}N$ HSQC spectra of uniformly ^{15}N-labeled Taf14-YEATS recorded upon titration with the indicated peptides. The spectra are color coded according to the protein:peptide molar ratio. Related to Figure 1.

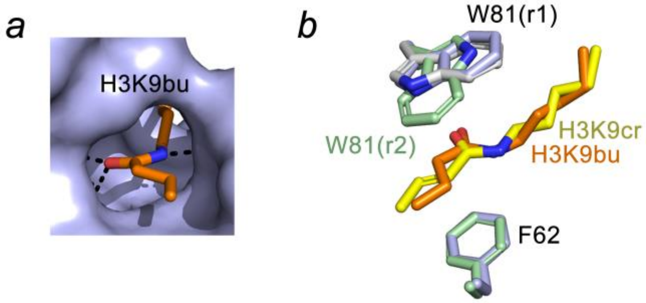

Supplementary Figure 3. (a) Close up view of the H3K9bu-binding tunnel of Taf14-YEATS. (b) Structural overlay of the acyllysine binding sites in the Taf14-YEATS:H3K9bu (lavender/orange) and Taf14-YEATS:H3K9cr[1] (light green/gray/yellow) complexes. Related to Figure 1.

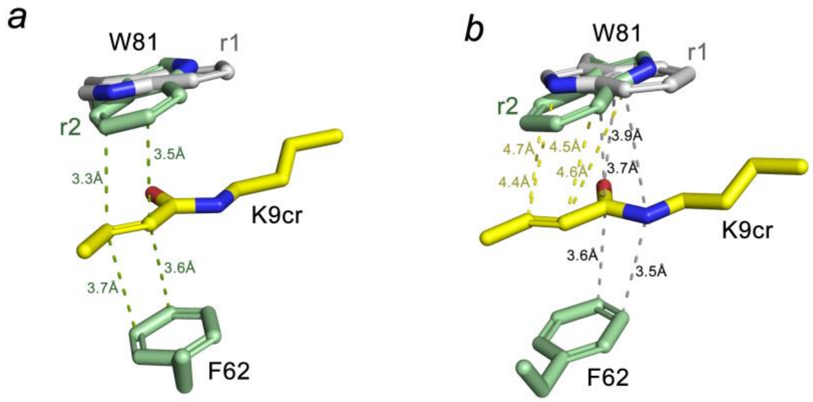

Supplementary Figure 4. Crotonylated lysine (yellow) is sandwiched between W81 and F62 in the complex of Taf14-YEATS with H3K9cr. W81 adopts two conformations, rotamer 1 (light gray) and rotamer 2 (green). (a) Green dashed lines represent short distances indicative of the aromatic-aliphatic-aromatic π stacking interaction for the r2 rotamer of W81. (b) Yellow dashed lines represent distances between the crotonyl alkene group and the aromatic group of W81 in the r1 conformation. Gray dashed lines represent short distances indicative of the aromatic-amide-aromatic π stacking interaction for the r1 rotamer of W81. Related to Figure 3.

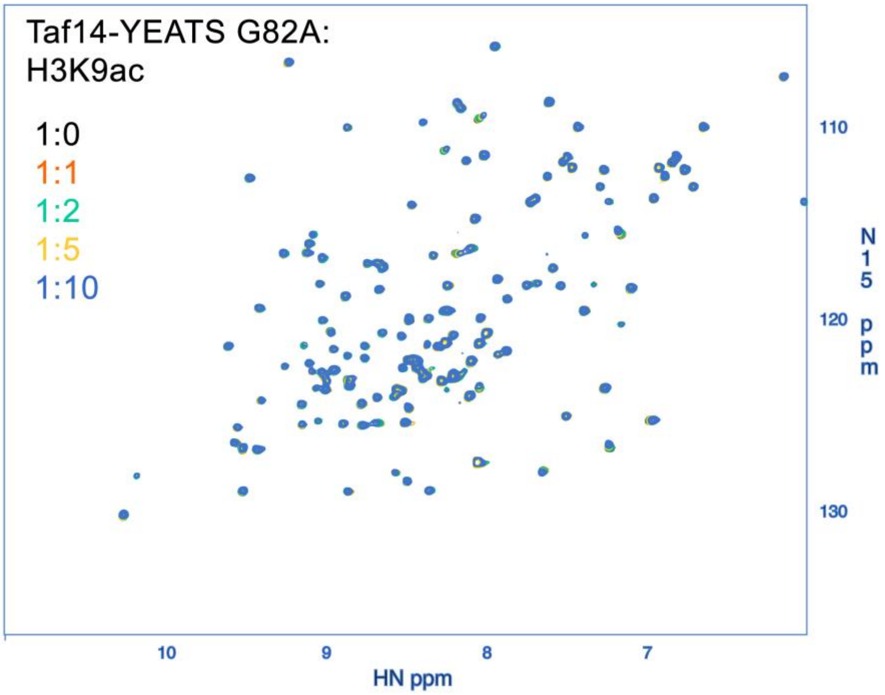

Supplementary Figure 5. Superimposed ^{1}H,^{15}N HSQC spectra of Taf14-YEATS G82A upon titration with of H3K9ac peptide. Related to Figure 2.

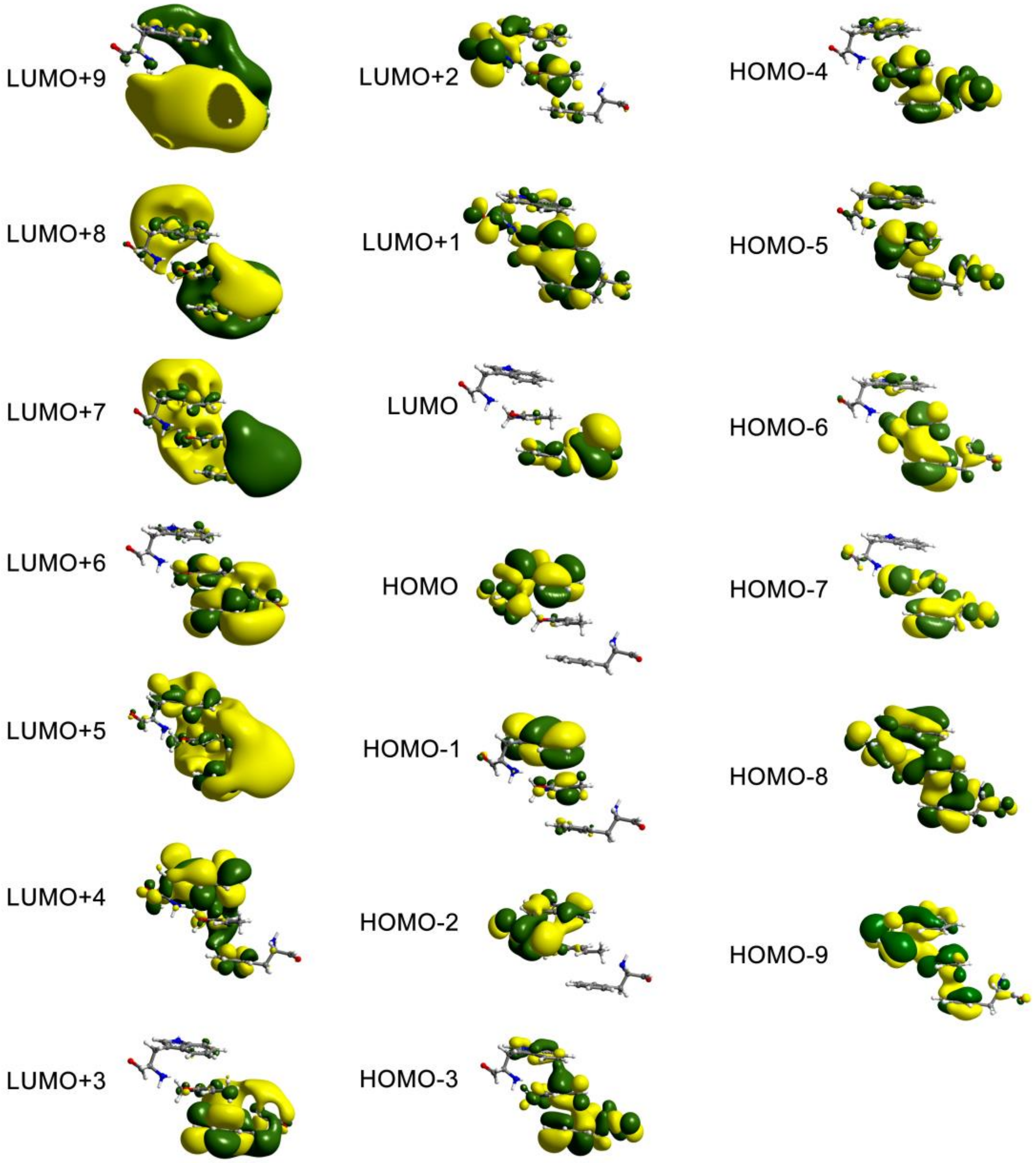

Supplementary Figure 6. MOs calculated in the W81, Kcr and F62 assembly by the B3LYP/6-311+G(d,p) level of DFT. Related to Figure 3.

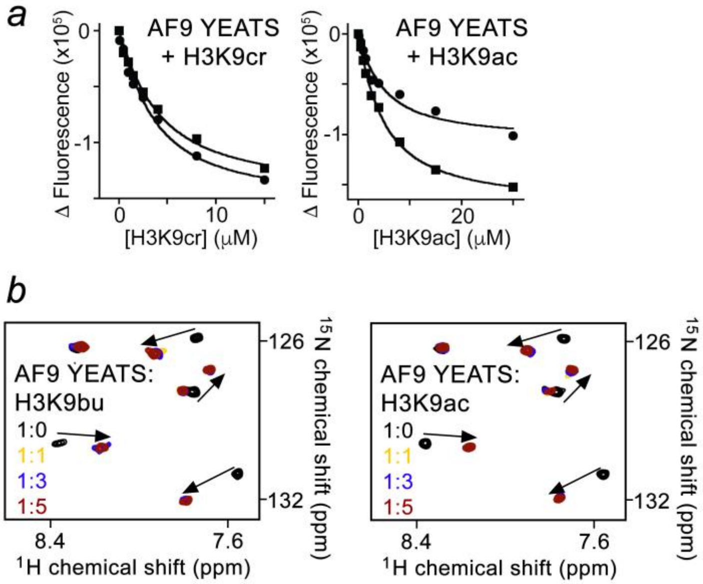

Supplementary Figure 7. (a) Binding curves used to determine K_d values of AF9-YEATS by tryptophan fluorescence. (b) Superimposed ^1H,^{15}N HSQC spectra of AF9-YEATS collected upon titration with the indicated peptides. Related to Figure 4.

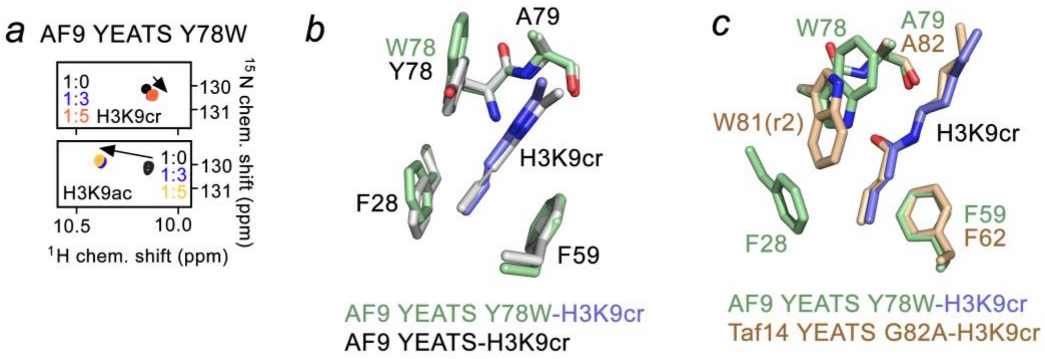

Supplementary Figure 8. (a) Superimposed ^{1}H,^{15}N HSQC spectra of AF9-YEATS Y78W collected upon titration with the indicated peptides. (b) Structural overlay of the acyllysine binding sites in the AF9-YEATS Y78W:H3K9cr (green/blue) and AF9-YEATS:H3K9cr[2] (light gray) complexes. (c) Structural overlay of the acyllysine binding sites in the AF9-YEATS Y78W:H3K9cr (green/blue) and Taf14-YEATS G82A:H3K9cr (wheat) complexes. Related to Figure 4.

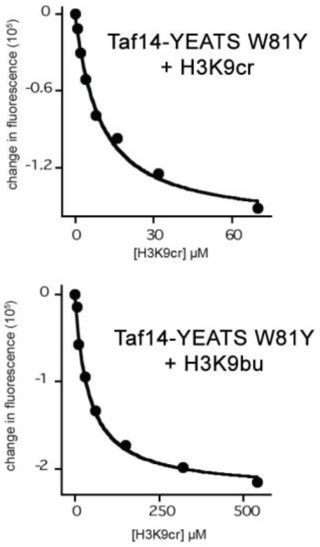

Supplementary Figure 9. Representative binding curves used to determine K_d values of Taf14-YEATS W81Y by tryptophan fluorescence. Related to Figure 5.

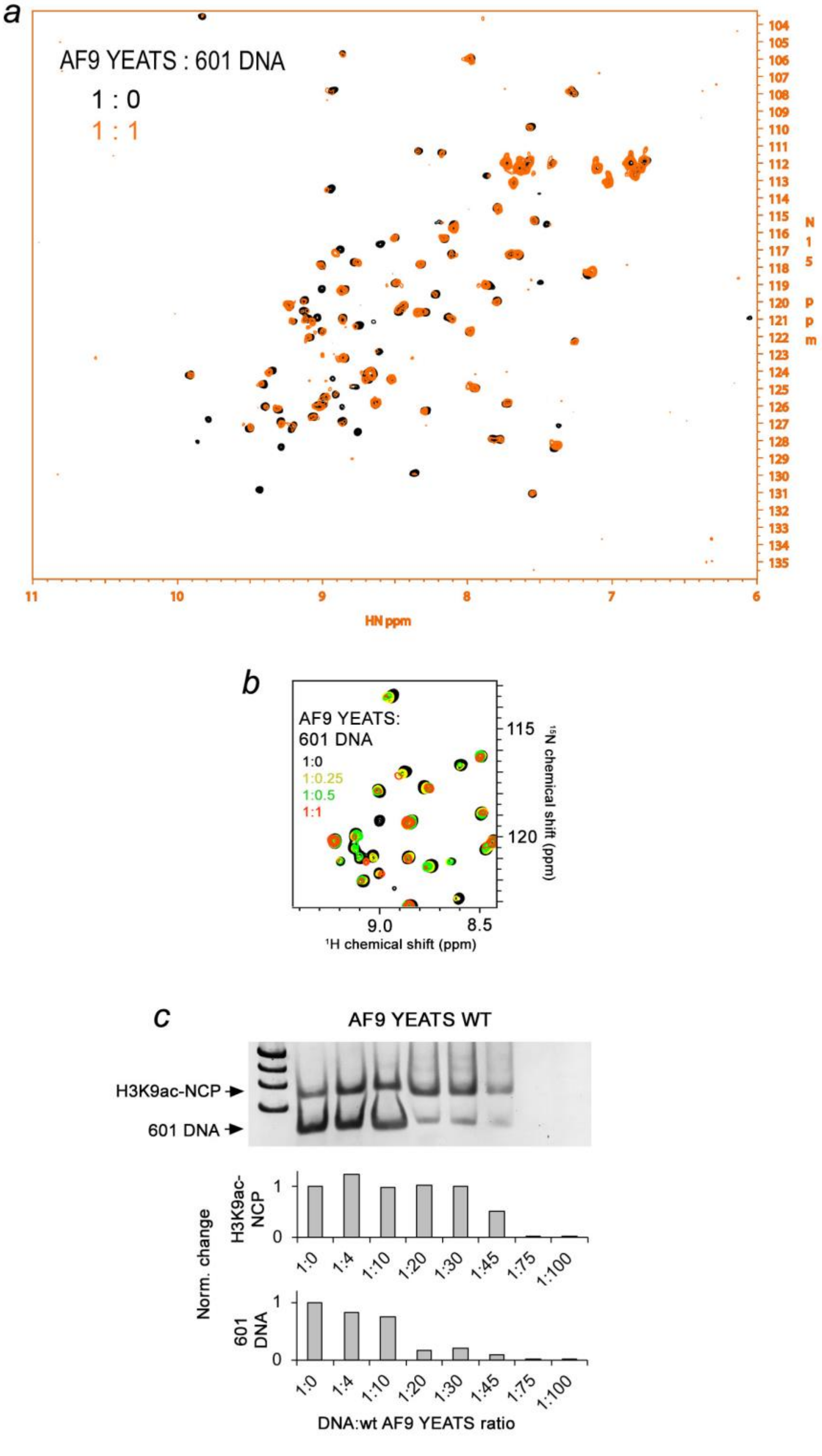

Supplementary Figure 10. (a, b) Superimposed ^{1}H,^{15}N HSQC spectra of AF9-YEATS upon titration with 601 DNA. Related to Figure 5. (c) EMSA with 1 pmol/lane H3K9ac-NCP incubated with increasing amounts of AF9-YEATS. Band intensities were quantified by densitometry using ImageJ. Related to Figure 5.

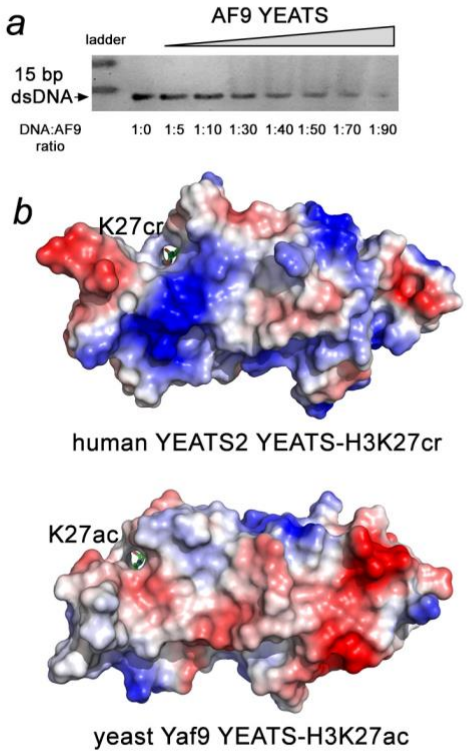

Supplementary Figure 11. (a) EMSA with 5 pmol/lane 15 bp-dsDNA incubated with increasing amounts of AF9-YEATS. (b) Electrostatic surface potential of the human YEATS2 YEATS domain in complex with H3K27ac peptide[3] (PDB ID 5XNV) or yeast Yaf9 YEATS domain in complex with the H3K27ac peptide[4] (PDB ID 6AXJ), generated in PyMol. Basic residues are shown in blue and acidic residues are shown in red. Related to Figure 7.

Primer Sequences

Taf14-YEATS W81F
5'-ctaaaggaaatccaccataaccttgttcctcaattctaaatggtgggt-3'
5'-acccaccatttagaattgaggaacaaggttatggtggatttcctttag-3'

Taf14-YEATS G82A
5’-gaggaacaaggttgggctggatttcctttagatataagc-3’
5’-gcttatatctaaaggaaatccagcccaaccttgttcctc-3’

AF9-YEATS Y78W
5'-ttggcaaaatgaaaccagcccacccagattcttctactttgtaagg-3'
5'-ccttacaaagtagaagaatctgggtgggctggtttcattttgccaa-3'

AF9-YEATS Y78W A79G
5’-tggcaaaatgaaaccaccccacccagattcttctactttgtaaggtgga-3'
5'-tccaccttacaaagtagaagaatctgggtggggtggtttcattttgcca-3'

AF9-YEATS R61E/K63E/K67E (RKK)
5’-cttgcacgaaagctttcctgagccagagagagtgtgcgaggatccaccttacaaagtag-3’
5’-ctactttgtaaggtggatcctcgcacactctctctggctcaggaaagctttcgtgcaag-3’

AF9-YEATS K92E
5’-gccaattgaagtttattttaaaaacgaggaagaacctaggaaagtc-3’
5’-gactttcctaggttcttcctcgtttttaaaataaacttcaattggc-3’

AF9-YEATS R96E/K97E
5’-gtttattttaaaaacaaggaagaacctgaggaggtccgctttgattatgacttattcctg-3’
5’-caggaataagtcataatcaaagcggacctcctcaggttcttccttgtttttaaaataaac-3’

AF9-YEATS R133E/K134E/K137E
5’-accccacagaggactttagggaggagttgctggaggcaggatagctcgagatc-3’
5’-gatctcgagctatcctgcctccagcaactcctccctaaagtcctctgtggggt-3’

Supplementary Figure 12. List of primers.

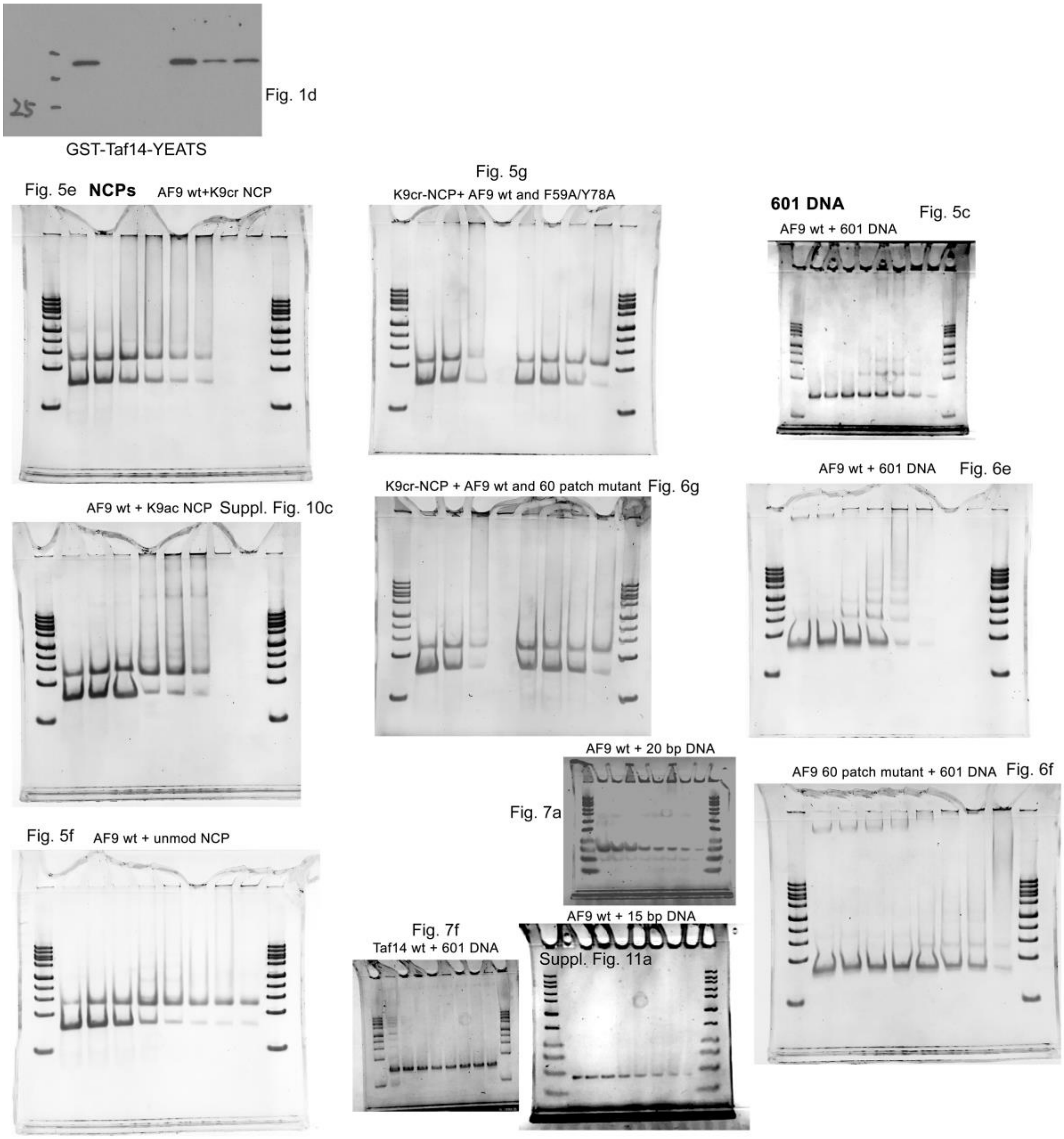

Supplementary Figure 13. Uncropped gels and Western blot.

Supplementary Table 1. Data collection and refinement statistics (molecular replacement)

	**Taf14 wt H3K9bu**	**Taf14 wt H3K9pr**	**Taf14 G82A apo**	**Taf14 G82A H3K9cr**	**AF9 wt H3K9bu**	**AF9 Y78W H3K9cr**
**Data collection**						
Space group	*P65*	*P65*	*P65*	*P65*	*C121*	*P212121*
Cell dimensions						
a, *b*, *c* (Å)	113.2, 113.2, 26.2	113.2, 113.2, 26.2	113.4, 113.4, 26.2	113.4, 113.4, 26.4	123.99, 44.9, 70.8	42.1 59.7, 155.4
α, β, γ (°)	90.0, 90.0, 120.0	90.0, 90.0, 120.0	90.0, 90.0, 120.0	90.0, 90.0, 120.0	90.0, 95.5, 90.0	90.0, 90.0, 90.0
Resolution (Å)	1.75 (1.81-1.75)*	1.85 (1.88-1.85)*	2.00 (2.03-2.00)*	1.90 (1.93-1.90)*	1.93 (2.0-1.93)*	2.53 (2.62-2.53)*
R_{sym} or R_{merge}	4.8 (50)*	5.4 (15)*	4.2 (9.2)*	4.8 (16.4)*	8.8 (40.7)*	9.9 (41.5)*
$I / \sigma I$	34 (5)*	51.8 (12)*	35.9 (18)*	40.0 (10.5)*	17.2 (1.5)*	33.5 (2.0)*
Completeness (%)	99.9 (99.9)*	97.8 (96.4)*	92.8 (93.7)*	96.7 (99.7)*	92.4 (63.3)*	94.3 (66.7)*
Redundancy	10.9 (11.2)*	4.3 (2.9)*	3.3 (3.8)*	4.3 (4.7)*	6.1 (2.7)*	10.8 (3.9)*
**Refinement**						
Resolution (Å)	56.6-1.75	37.1-1.85	37.1-2.00	37.0-1.90	48.8-1.93	47.3- 2.53
No. reflections	19812	15948	12217	14635	25016	12794
R_{work} / R_{free} (%)	17.3/21.8	18.2/23.2	18.0/21.8	20.2/25.6	18.3/23.6	20.3/26.4
No. atoms	1421	1390	1288	1337	2814	2547
Protein/peptide	1212	1207	1135	1165	2458	2382
Water	206	180	146	155	342	137
Ligand/ion	3	3	7	17	14	27
B-factors (Å^2)	31.07	26.13	31.65	30.56	38.0	46.45
Protein	29.14	24.81	30.53	29.33	37.84	46.64
Ligand/ion	28.84	24.04	47.53	52.19	71.17	43.27
Water	42.46	35.02	39.62	37.45	37.95	43.82
R.m.s. deviations						
Bond lengths (Å)	0.006	0.007	0.007	0.006	0.007	0.011
Bond angles (°)	0.91	0.91	0.89	0.87	0.93	1.29
Ramachandran quality						

Favored (%)	98.58	99.30	98.54	97.84	98.21	95.26
Allowed (%)	1.42	0.70	1.46	2.16	1.79	4.74
Outliers (%)	0	0	0	0	0	0

Supplementary References:

1. Andrews, F.H. et al. The Taf14 YEATS domain is a reader of histone crotonylation. *Nature Chemical Biology* **12**, 396-U33 (2016).
2. Li, Y. et al. Molecular Coupling of Histone Crotonylation and Active Transcription by AF9 YEATS Domain. *Mol Cell* **62**, 181-93 (2016).
3. Mi, W. et al. YEATS2 links histone acetylation to tumorigenesis of non-small cell lung cancer. *Nat Commun* **8**, 1088 (2017).
4. Klein, B.J. et al. Yaf9 subunit of the NuA4 and SWR1 complexes targets histone H3K27ac through its YEATS domain. *Nucleic Acids Res* **46**, 421-430 (2018).
